# Dr. Bogues Improvement upon the Kingsley Artificial Palate

**Published:** 1866-04

**Authors:** Geo. H. Cushing

**Affiliations:** Chicago, Ills.


### DR. E. A. BOGUE'S IMPROVEMENT UPON THE KINGSLEY ARTIFICIAL PALATE.

BY GEO. H. CUSHING, D.D.S., CHICAGO, ILLS.

For several months past, I have been looking for some notice in the *Register* or *Cosmos*, of Dr. E. A. Bogue's improvement upon the justly celebrated Kingsley's Artificial Palate. This improvement was perfected some time in May last, and yet, thus far, I think no notice of it has appeared in any of the dental journals. This, it seems to me, should not be so; as I conceive that an improvement so valuable to the profession, and to the public, should be duly accredited to its author; and as no other pen seems ready to move in this matter. I propose, briefly to state the chief points of merit which this improvement seems to claim, indulging the hope, that through the instrumentality of this article, the invention may come to be more generally used, and suffering mankind thus more largely benefited.

All are familiar, I presume, with the description of the original Kingsley Palate, beautiful and ingenious in its design, and successful in its application; but those who *are* familiar with it, know how complicated it was in its structure, and that very delicate and skillful manipulation was requisite in its construction; while it proved, upon experiment, to be open to the objection, that it became softened and unfit for use, after being worn for a few months, rendering its frequent renewal necessary. Dr. Bogue's improvement simplifies, very materially, the instrument, rendering it not so liable to get out of order; while by combining the hard with the soft rubber in its construction, together with its entirely different plan, it promises to do away, to a great degree at least, with the objection of softening.

The original Kingsley Palate, as all know, was made entirely of soft rubber, being made to fill completely the fissure, overlaping, both above and below, the muscles at the sides.

To enable the instrument to be fully effective, it was made capable of contraction and expansion, laterally. Contraction being effected by the action of the muscles upon it, while the expansion was produced by its elasticity, while the lower surface of it was on a continuous plane, from the anterior to the posterior margin.

The improvement of Dr. Bogue, consists in making the lower portion of the artificial palate, as far back as the posterior margin of the hard palate, of hard rubber, and the upper portion, of soft rubber; the upper or soft rubber portion extending back as far as might be desirable, to form a sort of apron, which lies closely upon and above the muscles of either side, and which is above the reach of the tongue. Thus the muscles are left free to act, by sliding *by*, and *under*, the soft rubber palate, not by contracting upon it. To all who are at all familiar with the construction of the original Kingsley Palate, if I have made my description at all clear, it will at once be apparent, how much simplified the instrument is, by Dr. Bogue's improvement. Its plan and principle are, in fact, entirely different from the original.

The softening which was found to occur in the original, was chiefly in the lower portion, which was in contact with the tongue; and it will appear, from the description of the improvement, that the tongue cannot come in contact with the soft rubber, to any great extent, as the anterior lower portion is made of hard rubber, while the soft palate proper, is carried above the reach of the tongue under ordinary conditions. Those put in after the improved method, thus far, have stood the test longer than any of the original style. Of course, time can only determine how long they will prove efficient without renewal; but one recently examined, which was inserted six months since, shows, as yet, no sign of softening.

As to the effectiveness of the improved palate, as compared with the original, I think it recommends itself; I have seen three cases in which the improved palate is worn, two of them made by Dr. Bogue, and one by Dr. J. C. Dean, of Chicago. In two of these cases, the Kingsley Palate had been previously adapted by Dr. Bogue; and the patients expressed themselves as much better pleased with the improved than with the original; stating that the new instrument was worn with greater comfort, as well as that it seemed to accomplish more perfectly, its intention than the old one.

Another improvement of Dr. Bogue's must not go unmentioned, and that is, the adapting the moulds to the Whitney Vulcanizing Flasks; an improvement, which those at all familiar with the process of making one of these palates, will not fail to appreciate.

The profession, and the cause of humanity, are largely indebted to Dr. Kingsley for his beautiful invention, and perhaps scarcely less so to Dr. Bogue, for his valuable improvements, which must eventually prove a great blessing to very many afflicted ones; and it is to be regretted that so few, thus far, have been benefited by the results of these gentlemen's labors in this direction. So far as I am aware, but three dentists, west of New York, have successfully applied the instrument—Dr. Bogue, Dr. J. C. Dean, of Chicago, and Dr. Moore, of Leavenworth, Kansas. Doubtless there are others who may have done so, but these are all that have come to my knowledge. Surely it seems desirable that so great a boon to those afflicted with cleft palate, should be more generally attainable than would seem to be the case. That it is not so, is doubtless due to the fact, that no sufficient instructions have yet reached the profession, generally, to induce them to undertake so difficult and delicate an operation; and it is to be hoped that Dr. Kingsley, and Dr. Bogue, both, will, very shortly, furnish to the profession, through the columns of the *Register* and *Cosmos*, full and explicit directions for the entire process; that those who desire, and have occasion, may be able to *undertake*, at least, to relieve such cases.

The making and adjusting properly, these artificial palates, requires a high degree of skill, will always be an expensive operation, and never one to be coveted for the mere pleasure of it; but there are many dentists, throughout the country, who would undertake it, and some successfully, in the hope of being able to relieve those suffering from "cleft palate," if they had full and clear instructions, and thereby many more would be benefited than can now hope to be.

I fear I have not done full justice to Dr. Bogue's improvements, in my description, but perhaps he will give a more satisfactory account of them himself, as well as directions for performing the necessary operations.

My object in writing this article, was two-fold: to call the attention of the profession to what seems to me to be *very* valuable improvements (in justice to their author) upon an invention, the value of which, we can, as yet, by no means fully appreciate; and to induce, if possible, the writing of such articles, by Drs. Kingsley and Bogue, as will stimulate the profession generally, to extend the field to its broadest limit, wherein the beneficence of these valuable inventions may find demonstration. I have no doubt that these gentlemen will cheerfully act upon the suggestion, so soon as their attention is called to it.

